# The Japanese Medical Science Federation COVID-19 Expert Opinion English Version

**DOI:** 10.31662/jmaj.2021-0002

**Published:** 2021-04-08

**Authors:** Masaomi Nangaku, Takashi Kadowaki, Hiroshi Yotsuyanagi, Norio Ohmagari, Moritoki Egi, Junichi Sasaki, Tetsuya Sakamoto, Yoshinori Hasegawa, Takashi Ogura, Shigeru Chiba, Koichi Node, Ryo Suzuki, Yasuhiro Yamaguchi, Atsuko Murashima, Norihiko Ikeda, Eriko Morishita, Kenji Yuzawa, Hiroyuki Moriuchi, Satoshi Hayakawa, Daisuke Nishi, Atsushi Irisawa, Toshiaki Miyamoto, Hidetaka Suzuki, Hirohito Sone, Yuuji Fujino

**Affiliations:** 1 The COVID-19 expert opinion committee of the Japan Medical Science Federation; 2 The Japan Medical Science Federation; 3 Japanese Association for Infectious Diseases; 4 Japanese Society of Intensive Care Medicine; 5 Japanese Association for Acute Medicine; 6 Japanese Society of Internal Medicine; 7 Japanese Respiratory Society; 8 Japanese Society of Hematology; 9 Japanese Circulation Society; 10 Japan Diabetes Society; 11 Japan Geriatrics Society; 12 Japan College of Rheumatology; 13 Japan Surgical Society; 14 Japanese Society on Thrombosis and Hemostasis; 15 Japan Society for Transplantation; 16 Japan Pediatric Society; 17 Japan Society of Obstetrics and Gynecology; 18 Japanese Society of Psychiatry and Neurology; 19 Japan Gastroenterological Endoscopy Society; 20 Japan Society for Occupational Health; 21 Japan Epidemiological Association; 22 Japanese Society of Respiratory Care Medicine

**Keywords:** COVID-19, SARS-CoV-2, coronavirus, infection control, respiratory management

## Abstract

In 2020, the COVID-19 pandemic has had unprecedented impacts on various aspects of the world. Each academic society has published a guide and/or guidelines on how to cope with COVID-19 separately. As the one and only nationwide association of academic societies that represent medical science in Japan, JMSF has decided to publish the expert opinion to help patients and care providers find specifically what they want.

This expert opinion is a summary of recommendations by many academic societies and will be updated when necessary. Patients that each academic society targets differ even though they suffer from the same COVID-19, and recommendations can be different in a context-dependent manner. Readers are supposed to be flexible and adjustable when they use this expert opinion.

## Presidential Message

The Japanese Medical Science Federation (JMSF) is the sole comprehensive association of medical academic societies representing the medical science field of Japan. It aims to contribute to the development of the quality of medical research and medical practices by promoting medical science and technology and setting a high-compliance standard of conduct among medical researchers. It now comprises 136 academic societies: 103 in clinical medicine, 19 in social medicine, and 14 in basic medicine. The number of members of these societies is over 1 million. By playing an important cross-cutting role among those medical societies, JMSF assumes the responsibility of promoting the health of people.

In 2020, the COVID-19 pandemic has had unprecedented impacts on various aspects of the world. We as healthcare providers and medical researchers have devoted ourselves to combat COVID-19. Each academic society has published a guide and/or guidelines on how to cope with COVID-19; however, we hear that it is rather difficult for patients and care providers to find specifically what they want, since these guidelines are being published and distributed separately. The philosophy and basic principle of JMSF is that medicine and medical practice should protect the healthy lives of individual citizens and groups in society, so as to contribute to the welfare and well-being of all people. Thus, as the one and only nationwide association of academic societies that represent medical science in Japan, JMSF has decided to address these public needs.

As the president of JMSF, I asked Dr. Takashi Kadowaki, Vice President of JMSF, and Dr. Masaomi Nangaku, Chairman of the Guideline Committee of JMSF, to compile this “expert opinion” to meet many requests from various people.

I firmly believe that this “expert opinion” will be of great use to you.

President of JMSF

Morito Monden,

M.D., Ph.D.

## How to Use This Expert Opinion

Under the leadership of President Morito Monden and Vice President Takashi Kadowaki, the Guideline Committee concluded the following:

(1) Early publication is necessary.

(2) As COVID-19 is a new clinical entity, we cannot make guidelines based on robust clinical evidence.

(3) It is desirable to publish “expert opinion” and revise it in real time when new clinical evidence is obtained.

(4) It must be concise for the sake of readability.

According to this policy, JMSF asked academic societies to nominate their representatives and successfully compiled this expert opinion in a short time. I sincerely thank all the members of this working group for their devotion. Moreover, I would like to emphasize the huge contribution of the members of the Japanese Association for Infectious Diseases, the Japanese Society of Intensive Care Medicine, and the Japanese Association for Acute Medicine.

Please note that this expert opinion is a summary of recommendations by many academic societies. Patients that each academic society targets differ even though they suffer from the same COVID-19. Thus, recommendations can be different in a context-dependent manner. This difference reflects the diversity of patients of COVID-19. Please be flexible and adjustable when you use this expert opinion.

JMSF

Chair of COVID-19 Expert Opinion Working Group

Masaomi Nangaku

## Outpatient Clinics

### Infection control

Clinical courses of COVID-19 are mild in 80% of patients. The mortality of COVID-19 is reported to be higher than that of seasonal influenza. The risk of severe disease is especially high in the elderly; those with underlying disease such as chronic obstructive pulmonary disease, chronic kidney disease, diabetes, hypertension, cerebrovascular disease, cardiovascular disease, and obesity; pregnant patients; those with active malignancy; and those with immune suppression ^(1)^.

Important measures against COVID-19 in general internal medicine are strict implementations of the standard prophylaxis of infection, prevention of aggravation of underlying disease, and appropriate lifestyle to prevent new risks of severe disease. The elderly should also prevent frailty by exercise and activities at home along with prophylaxis of infection because staying home to avoid infection may accelerate frailty ^(2)^.

The main infectious routes of SARS-CoV-2 are droplet infection* and contact infection**. In addition to droplets, aerosols in exhaled air are thought to be infective. Conversation at a closed distance in closed conditions expands infection. Infectivity is the strongest 2 days before and a few days after the onset of the disease. The incubation period after exposure to the source of infection is 1 to 14 days, and patients most often develop the disease about 5 days after exposure.

To prevent nosocomial infection, patients should make a phone call in advance when they come to an outpatient clinic. Telemedicine is also useful. Another method is to have an “outpatient clinic for patients with fever” by separating patients with fever from those who come to a general outpatient clinic. If it is difficult to separate patients with fever spatially, temporal separation can also be a choice. Patients with fever, respiratory symptoms, and history of exposure should be screened for the outpatient clinic for patients with fever. In case of a history of exposure only, patients should return home. If patients complain of symptoms, doctors should see them and decide the necessity of diagnostic examination for COVID-19. Preventive measures against droplets and contact in addition to standard preventive measures are essential at the outpatient clinic for patients with fever. If COVID-19 is suspected and generation of aerosol by sampling procedures is expected, one should employ measures to prevent airborne infection. Adequate ventilation is critical during a medical examination.

In the case of face-to-face medical examination, both medical staff and patients are required to wear masks. If the mask cannot be worn due to the patient’s conditions, medical staff will protect their eyes by additional personal protective equipment, such as eye shields, goggles, and face shields ^(3)^.

*Droplet infection: This is an infection transmitted from infected person to another by droplets (sneezing, coughing, spit, etc.) expelled from the upper respiratory tract.

**Contact infection: For example, if an infected person holds a sneezing, coughing, or runny nose with his/her hand and then touches surrounding objects with that hand, the surface will be contaminated with the virus. When others touch their mouths and noses after touching the surfaces contaminated with the virus, they can become infected through the mucosa.

***Cough etiquette: Use a mask, tissue, handkerchief, or sleeves to hold your mouth and nose when coughing or sneezing.

****Precautions for hand washing: Hands can be contaminated with virus by touching things many unknown persons have touched, such as door knobs or train hand straps. It is important to wash your hands thoroughly, for example, when you return home, before and after cooking, and before eating. If you cannot wash your hands with soap and running water, use alcohol-based disinfectants on the fingers.

### How to proceed with diagnosis and testing

Check the medical history and symptoms by phone or interview sheet in advance, check travel history to foreign countries and endemic areas and history of close contact with people from endemic areas and COVID-19 patients, and check duration of fever, cough, prevalence in the community, and loss of smell and taste. If you get inquiry by a patient with suspected COVID-19 and your institution does not do diagnostic tests of COVID-19, please refer to your regional institution that takes care of COVID-19 patients ^(4)^.

It is also important to perform a differential diagnosis of other infections. Under the endemic situation of other infections such as influenza, one needs to perform diagnostic tests of both seasonal influenza and COVID-19 at the earliest convenience ^(5)^. If the capacity of diagnostic tests of COVID-19 is limited, it is also reasonable to perform tests of influenza first and treat patients with positive tests ^(6)^.

Radiological examination of the chest and genetic examination of SARS-COV-2 are performed as diagnostic tests (check the chapter on “Hospitalization”).

A doctor who has made a diagnosis of COVID-19 should immediately report to a regional public health center.

### Concerning drugs for chronic diseases

The risk of RAS inhibitors to COVID-19 is not beyond theoretical concerns, and there is no clear evidence for recommending discontinuation or change to other drugs. Therefore, they should be used in line with conventional indications.

Data on how immunosuppression affects the risk of infection and aggravation of COVID-19 are limited. Considering the risk of relapse or exacerbation of the underlying disease due to dose reduction or discontinuation, immunosuppressants, biologics, anti-rheumatic drugs, and glucocorticoids should be continuously administered at the same dose in principle ^(7)^.

If there are signs of infection, maintain the same dose of steroids in principle and carefully reduce the dose of biologics and immunosuppressants or temporarily postpone administration. Careful practices are needed in the same way as for ordinary infections.

## Emergency Department

There is a risk of encountering a variety of human-to-human infections, including emerging infections, in the emergency department, and adequate and appropriate measures must be taken to prevent infection. “COVID-19 infection cannot be ruled out in all patients.” However, guidelines based on sufficient evidence for infection control in the emergency department have not been developed worldwide, and each institution is currently examining and implementing its own countermeasures.

Infection control in emergency medicine has the following characteristics:

A. It is necessary to perform highly invasive and rapid medical care in parallel.

B. The content of this is diverse, such as preventive measures by infection route, disinfection management of medical equipment and environment, surveillance, surveillance for drug-resistant bacteria, including proper use of antimicrobials, etc.

C. Collaboration between ER staff and infection control staff, such as ICT, is also important, and the perspective of “multidisciplinary team medicine” is necessary.

The following is useful reference information related to emergency care.

### Checklist for infection control in the emergency department

The Committee for Infection Control in the Emergency Department and The Joint Working Group (Japanese Association for Acute Medicine, Tokyo, Japanese Society for Infection Prevention and Control, Tokyo, Japanese Association for Infectious Diseases, Tokyo, Japanese Society for Emergency Medicine, Tokyo, and Japanese Society for Clinical Microbiology)

This checklist was developed based on a comprehensive and multifaceted review of infection control and related issues in the emergency department ^(8)^ in order to ensure that even small emergency departments with few or no dedicated emergency physicians can prepare for infection control without making major mistakes if they follow this checklist. The checklist includes a management system for infection control, education, screening, and vaccination system, handling of suspected infected patients, and hardware for infection risk management, and the timing and intervals between checks are clearly indicated as categories.

### Countermeasures for cardiopulmonary arrest (CPA) cases (including prehospital care) with novel coronavirus infections (COVID-19)

The Committee for Infection Control in the Emergency Department and The Joint Working Group (Japanese Association for Acute Medicine, Tokyo, Japanese Society for Infection Prevention and Control, Tokyo, Japanese Association for Infectious Diseases, Tokyo, Japanese Society for Emergency Medicine, Tokyo, and Japanese Society for Clinical Microbiology)

Although airborne infection prevention measures are not generally required at the time of CPA cases in the emergency department, consideration should be given to reducing the risk of exposure of medical personnel to infection from aerosol-generating procedures in light of the current spread of novel coronavirus infections. From the point of view of “practicable and concrete proposals that can be implemented in response to reality” including the prevention of unnecessary waste of medical treatment materials, the basic ideas for medical examinations are presented ^(9)^.

The basic approaches for CPA cases, including prehospital care, are as follows:

(a) If neither fever nor respiratory symptoms can be ruled out prior to cardiac arrest

- Make usual responses with standard personal protective equipment (PPE).

(b) If an episode of fever or respiratory symptoms prior to cardiac arrest can be heard

- Add the N95 mask to the usual PPE that covers the eyes, nose, and mouth (surgical mask with eye shield or surgical mask with goggles/eye shield/face guard combination), gown, and gloves.

(c) Insufficient information about fever and respiratory symptoms before cardiac arrest

- Make a comprehensive judgment based on the presumed cause of cardiac arrest, the prevalence of the disease in the region, and the supply and demand for N95 masks and other products.

### Heatstroke management during the COVID-19 epidemic: recommendations from the experts in Japan

Working group on heatstroke medical care during the COVID-19 epidemic (Japanese Association for Acute Medicine, Japanese Society for Emergency Medicine, Japanese Association for Infectious Diseases, Japanese Respiratory Society)

Since fever and hyperthermia are the main symptoms of both COVID-19 and heatstroke, it is difficult to differentiate them. In the new lifestyle, such measures as adequate indoor ventilation, wearing masks, and ensuring physical distance between people are some of the issues to be taken into account in heatstroke prevention, thereby providing a guide for the prevention of summer heatstroke in the so-called corona crisis ^(10)^.

### Manual of cardiopulmonary resuscitation in hospitals for novel coronavirus infection (COVID-19)

#### Japan resuscitation council (JRC)

This manual aimed to provide rapid and safe cardiopulmonary resuscitation in hospitals, and it is primarily intended for those who are in charge of first aid and emergency care in hospitals ^(11)^. The contents of this manual are general principles and may need to be adjusted according to the local infection situation because this is a supplementary resuscitation manual developed from the perspective of protecting medical personnel in hospitals from infection. For reference, major guidance in Europe and the United States is listed below.

(a) International Liaison Committee on Resuscitation. COVID-19 Practical Guidance for Implementation ^(12)^

(b) Emergency Cardiovascular Care Committee and Get With The Guidelines-Resuscitation Adult and Pediatric Task Forces of the American Heart Association: Interim Guidance for Basic and Advanced Life Support in Adults, Children, and Neonates With Suspected or Confirmed COVID-19 ^(13)^

(c) American Heart Association: Interim Guidance for Healthcare Providers Caring for Pediatric Patients ^(14)^

(d) European Resuscitation Council: European Resuscitation Council COVID-19 Guidelines ^(15)^

(e) Resuscitation Council (UK): Statements and Resources on COVID-19 (Coronavirus), CPR, and Resuscitation ^(16)^

### Guide to resuscitation training course during the novel coronavirus infection (COVID-19) pandemic

#### Japan resuscitation council (JRC)

The resuscitation training course is essential, considering its significance in saving the lives of cardiac arrest victims. We should protect medical personnel and citizen participants from COVID-19 through the training course with appropriate infection control measures. This is a guide to hold a safe training course during the COVID-19 epidemic period ^(17)^.

## Hospitalization: Internal Medicine

### Infection control

Before and during admission, one should confirm symptoms, exposure to patients with COVID-19 within the last 2 weeks, history of foreign travel, and history of going to facilities where infection clusters are reported. If one of the above is positive, re-evaluate necessity of examination of COVID-19 and preventive measures in the hospital.

If patients have fever, the standard preventive measure should be employed if there are other diseases that are likely to be the cause of the fever. If not, re-evaluate the necessity of hospitalization. As in outpatient clinics, hospitalized patients must also wear masks. If patients cannot wear masks, medical staff should protect their eyes.

For informed assent during hospitalization, use a large room with good ventilation for explanation to patients and family members with attention to privacy. The time of explanation should be short, and if it takes long, ventilation during the explanation may help.

### How to proceed with diagnosis and testing

Based on the patient’s clinical symptoms (e.g., fever, respiratory symptoms including mild symptoms, loss of smell and taste), history of close contact with COVID-19 patient, prevalence in the community, and lifestyle (travel history, eating and drinking, and participation in events), if the SARS-CoV-2 coronavirus disease (COVID-19) is suspected, diagnostic tests to confirm COVID-19 should be performed.

Chest X-ray findings have many variations, including no abnormalities observed. It is difficult to point out ground-glass opacities (GGOs) in the early phase of the disease, but they are useful for assessments in advanced stages or following up of the disease.

Chest computed tomography (CT) should not be used for the screening of low-risk and asymptomatic patients, but it can be useful in the assessment of high-risk patients and for specific purposes. It is important to know how many days have passed from the date of disease onset because the chest image findings could dramatically change depending on the severity of pneumonia in the onset or the advanced stage. In the early phase, subpleural GGOs are typically observed. As the disease progresses to severe, crazy-paving appearance with reticulation within the GGOs of the lobes and more infiltrative shadows may increase.

Genetic (RT-PCR, LAMP, etc.) and antigen tests (quantitative and qualitative) are used to make a definitive diagnosis. The most reliable test is the PCR test, which has high sensitivity and specificity and is the gold standard for the diagnosis of COVID-19. Antigen quantitative tests and LAMP are also practical test methods. In addition, antigen quantitative testing can be used for symptomatic patients.

If the pretest probability is low, a test method with high sensitivity and specificity should be used. Each of these two tests would be required to collect samples at the appropriate time (refer to the Ministry of Health, Labour and Welfare, “Guidelines for testing for novel coronavirus (COVID-19) pathogens (second edition),” Table 3, Figure 2).

Antibody testing is not conducted as an administrative test and is not designated as a test for definitive diagnosis.

### Measures for hospitalized patients who develop fever or contract infectious diseases

Standard preventive procedures will be implemented, and patients will immediately undergo a close examination following the standard procedure for fever onset. Cases wherein pneumonia is suspected based on imaging findings, or the clinical course is atypical, will be managed as suspected COVID-19: Isolation measures will be implemented, and then diagnostic testing will be performed (e.g., PCR or antigen test). When a patient is confirmed to have COVID-19, persons in close contact with the patient will be identified, and cohorting will be implemented.

### Procedures for COVID-19 treatment

Disease status is classified into four stages: mild, asymptomatic carrier or coughing only; moderate I, pneumonia and/or breathlessness but not respiratory insufficiency; moderate II, respiratory insufficiency requiring supplemental oxygen; and severe, respiratory failure requiring ventilation or ICU admission.

Even when mild COVID-19 is initially diagnosed, the patient’s condition can progress over the course of 7-10 days: Careful observation is required, especially for patients with risk factors such as old age, underlying disease (e.g., diabetes, heart failure, COPD, hypertension, and cancer), immunosuppression, pregnancy, and obesity.

When supplemental oxygen is required, such as when oxygen saturation (SpO2) is less than 94% on room air, medication including antiviral agents will be considered in addition to symptomatic therapy. Such medication is suggested for patients whose condition is classified as moderate I (requiring no supplemental oxygen) with a risk factor for disease progression, but there is still no clear evidence.

Severe disease and respiratory failure are moderate risk factors of arterial and venous thrombosis (e.g., deep vein thrombosis), and anticoagulant therapy (e.g., heparin) will be provided when the D-dimer level exceeds the upper limit of the normal range.

Severe cases require highly specialized treatment, such as tracheal intubation, extracorporeal membrane oxygenation (ECMO), and blood purification. Specialist knowledge of intensive care and monitoring systems is necessary to implement such treatment properly.

#### Steroids

Improvement of prognosis by steroids has been confirmed in patients with moderate II or severe COVID-19. Treatment with dexamethasone (6 mg) for 7-10 days showed the highest level of evidence. Other agents with equivalent potency can be used as alternatives, such as prednisolone (40 mg) and methylprednisolone (32 mg). On the other hand, improvement of prognosis by steroids has not been demonstrated in patients without the need for supplemental oxygen; instead, the use of steroids may worsen prognosis. Therefore, the use of steroids is not recommended for these patients. Nevertheless, oxygenation of COVID-19 patients can become impaired over time, and careful observation is necessary. When switching the administration route between oral and intravenous injections, equipotent doses will be administered.

#### Approved antiviral agents in Japan

Remdesivir, which has been granted special approval in Japan, is the only antiviral agent for treating COVID-19 (as of November 2020). It is indicated for patients requiring supplemental oxygen, and patients should be carefully monitored after administration of remdesivir because acute kidney or liver damage may occur. The administrative procedure (completing a web-based survey) specified by the Ministry of Health, Labour and Welfare ^(18)^ must be followed to obtain remdesivir.

#### Other antiviral agents and agents that have not yet been approved in Japan

Although their use for the treatment of COVID-19 has not yet been approved, favipiravir, tocilizumab, ciclesonide, nafamostat, sarilumab, nelfinavir, ivermectin, and convalescent plasma are sometimes considered for off-label use or for use based on the findings of investigator-initiated or industry-sponsored clinical studies.

### Ventilation management

Due to the high possibility that aerosols will be generated during endotracheal intubation, practitioners should wear a N95 mask and PPE that covers their eyes, neck, and skin. Using respiratory devices covered by a surgical mask reduces the risk of nosocomial infection in healthcare workers. However, a negative pressure room and PPE that includes the N95 mask and eye protection should be used when performing respiratory management, which can generate aerosols. The use of noninvasive positive pressure ventilation and high-flow nasal cannula may have a potential for aerosol dispersion, but at this time, there is no sufficient consensus on the use of these techniques ^(19)^.

### Consideration for patients receiving chemotherapy or those in immunodeficiency condition when complicated with COVID-19

If possible, chemotherapy or immunosuppressive therapy should be discontinued and treatment for COVID-19 performed. Due to the high risk of exacerbation, blood oxygen saturation is frequently monitored. Neutropenic conditions increase mortality risk. In addition, it has been reported that mortality is higher in blood cancer-bearing patients in non-remission than in remission and in those receiving chemotherapy containing steroids and anti-CD20 antibodies than in those receiving chemotherapy without these drugs.

## Hospitalization: Surgical Treatment

Identifying asymptomatic or minor symptomatic SARS-CoV-2 carriers by interview and general examination is difficult. If surgery is performed under general anesthesia in patients with subclinical infection, serious postoperative complications from COVID-19 may occur, in addition to the risk of nosocomial infection. It is recommended that PCR testing be performed to screen patients who are awaiting surgery, and surgery should be performed after negative test results are confirmed. Even in asymptomatic patients, if the physician determines that screening is necessary, it will be covered by insurance in Japan ^(20)^.

On the other hand, personnel should be made aware that tracheal intubation, extubation, electrocautery, ultrasonic coagulation, and other energy devices during surgery and laparoscopic surgery can generate aerosols, which can increase the risk of droplet infection. In addition to wearing PPE, high-precision filters and flue gas devices are recommended for intraoperative infection control ^(21)^.

### Preoperative diagnosis/examination

#### 1) Radiological examination

Examinations for screening purposes can be postponed. If a disease is strongly suspected or there is a need to determine treatment methods, the examination should be performed as usual. However, in the event that chest X-ray or chest CT scan shows ground-glass opacity or infiltrative shadows and COVID-19 infection is suspected, the hospital protocol should be followed, and corresponding departments should be consulted.

#### 2) Endoscopy

Considering the spread of infection and the patient’s condition, it is recommended that non-emergency endoscopies be postponed. In the gastrointestinal field, examinations should be conducted if the patient presents with bleeding, symptoms of dysphagia, obstructive jaundice, etc., or strong suspicion of malignancy, or if examination is deemed necessary.

Since both gastrointestinal endoscopy and bronchoscopy carry the risk of infection due to aerosol generation, refraining from testing is safer if COVID-19 infection cannot be excluded. However, if it is to be conducted, use of PPE by healthcare professionals should be strictly adhered to.

For more information, refer to the Endoscopy section.

### Surgery

In a situation where COVID-19 infection has spread, a negative impact on surgical treatment may be inevitable due to limitations in the medical system, which may occur as a result of the shift in practice. The decision to treat the primary disease under these circumstances, in addition to ensuring patient safety, prevention of exposure of medical personnel, including surgeons, and the prevention of nosocomial infection must all be met. When postponing or canceling non-urgent surgery (surgical triage), the condition of the patient, severity of the disease, local infection status, and medical supply situation (medical resources, human resources, and medical equipment) at each facility should be comprehensively considered ^(21)^, ^(22)^. The “Recommendations for surgery for patients with confirmed or suspected COVID-19 infection” ^(23)^ have reached a consensus among surgical societies and are indicative.

Surgery can be postponed in cases of benign disease with minor symptoms or early stage cancer that can wait for treatment on a monthly basis; however, surgery should be performed with appropriate infection prophylaxis in cases of advanced cancer or cases requiring semi-urgent treatment (Table 1).

**Table 1.** Guideline for Performing Surgical Triage during the COVID-19 Pandemic ^(24)^.

Status of the medical care system			Stable period		Restricted period	
Status of COVID-19 on target patient			Negative	Positive or suspected	Negative	Positive or suspected
Disease level	A	Disease that is nonfatal or does not require urgent medical intervention	Perform cautiously under appropriate infection control	Postpone	Postpone	Postpone
	B	Disease that is nonfatal but may potentially be fatal or is at risk of becoming severe	Perform surgery cautiously under appropriate infection control	Postpone; if not possible, perform surgery cautiously under full infection control	Postpone if possible	Postpone
	C	Disease that may be fatal in a few days or a few months without any surgical intervention	Perform surgery cautiously under appropriate infection control	Consider alternative treatment options; if not possible, perform surgery cautiously under full infection control	Consider alternative treatment options; if not possible, perform surgery cautiously under appropriate infection control	Consider alternative treatment options; if not possible, perform surgery cautiously under full infection control

(Quated from Japan Surgical Society [Internet]. [cited 2021 Feb 1]. Available from: https://www.jssoc.or.jp/aboutus/coronavirus/info20200414.pdf. Japanese ^(24)^. Citation of this table is approved by the Japan Surgical Society).

With regard to patients who are expected to have difficulty in weaning from mechanical ventilators early after highly invasive surgery and who are expected to be in the ICU for an extended period of time, management of such patients should be considered by a multidisciplinary team.

Significant points for surgical treatment for COVID-19-infected patients are described by region (gastrointestinal tract, hepatobiliary, cardiovascular, general thoracic, breast, pediatric, and transplantation) in a publication of the Japan Surgical Society ^(21)^.

Furthermore, according to an international survey, it has been reported that patients infected with COVID-19, 7 days prior to surgery and 30 days after surgery, tend to develop severe conditions with a 23.8% mortality rate within 30 days, of which over 80% were due to pulmonary complications ^(25)^. Therefore, special attention must be paid to decision-making for surgery for patients who have risk factors, such as advanced age, smoking, multiple comorbidities, and pre-existing lung disease.

According to a large-scale surgical triage survey of 359 hospitals in 71 countries, including Japan, 73% of the surgeries (approximately 1.4 million surgeries: upper and lower gastrointestinal, hepatobiliary, urological, head and neck, gynecological, plastic, orthopedic, and obstetric) scheduled to take place over the 12-week period beginning after late March were canceled or postponed. Of these, there were about 98,000 and 1,253,000 cases of cancer (30%) and benign diseases (84%), respectively. It was estimated that it would take 45 weeks to clear the backlog of these canceled or postponed surgeries, even if the pre-COVID-19 pandemic surgical regime was strengthened by 20% ^(26)^. The Japan Surgical Society’s Proposals for Providing Surgical Care for the Convergence of the Coronavirus Infectious Pandemic suggests that the resumption of elective surgery during the COVID-19 lull period has reached a consensus among surgical societies and will act as a reference for each facility ^(27)^.

## Management of Visitors to Hospitalized Patients

The staff and any visitors from outside (family, friends, volunteers, students, entrusted personnel, etc.) can possibly bring virus to the patient’s room. Therefore, depending on the outbreak conditions in the local area or in the facility, it is sometimes necessary to restrict visitors from entering the patient’s room. However, careful consideration is necessary for family visit management, particularly when the patient is at the end stage. Psychological support to patients tends to decrease due to the visit restriction; compensatory care should be considered as much as possible.

When restricting contact between visitors and patients according to the director’s instructions, sufficient explanations are necessary to allow visitors to understand the situation. If visitor-patient contact is permitted, appropriate preparations for hand hygiene and mask wearing are required. As an alternative to direct contact, consider online systems using videophones, etc.

Body temperature should be measured of all visitors before admission. The admission of febrile persons should not be allowed. Recording visitors’ information can be considered for an active epidemiologic survey.

### Intensive Care and Respiratory Management

Among patients with COVID-19, the proportion of patients suffering severe COVID-19 requiring intensive care varies from about 20% from Europe and the United States to 7-9% in Japan, depending on the report. In any scenario, according to the increase in the number of COVID-19 patients, the number of severely ill COVID-19 patients requiring invasive treatments such as mechanical ventilation and ECMO would be increased.

To prevent the transmission of COVID-19 during intensive care, multidisciplinary healthcare workers must be proficient in the use of PPE to ensure their own safety while treating patients. Particularly during intubation and extubation, healthcare workers have a high risk of exposure to the virus due to aerosol generation; therefore, appropriate preventive measures must be taken. Please refer to the “COVID-19 Patient-Nursing Practice Guide (ver. 2)” ^(28)^.

When a patient with COVID-19 requires mechanical ventilation, it is important to provide a lung-protective strategy, avoiding high tidal volume and airway pressure. To minimize ventilation-related lung injury, it is also important to provide appropriate sedation, muscle relaxation, and prone therapy. Please refer to the “ARDS Practice Guidelines 2016” ^(29)^ for respiratory management in patients with COVID-19. Some precautions should be taken in the respiratory management for COVID-19 patients compared with usual respiratory management. Please refer to the “Precautions in the Ventilatory Management of Patients with COVID-19 (ver. 2)” ^(30)^.

In severe COVID-19 patients who are difficult to treat with mechanical ventilation, ECMO might be required. In this case, please refer to “Basic information on the management of mechanical ventilation and ECMO for COVID-19 acute respiratory failure” ^(31)^.

It is necessary to prevent the transmission of COVID-19 during management of mechanical ventilation. Please refer to the “Guide for management for COVID-19 patients requiring mechanical ventilation from the point of view of preventing transmission through medical devices (ver. 2)” ^(32)^.

Patients with moderate to severe COVID-19 might have not only acute respiratory failure but also acute myocardial dysfunction, acute kidney injury, shock, and coagulopathy. They would require intensive care therapies, similar to other critically ill patients. Until now, there has been no specific ICU management for patients with severe COVID-19. Therefore, intensive care should be performed according to other critically ill patients. Please refer to the Japanese Clinical Practice Guidelines for Management of Sepsis and Septic Shock 2020 (J-SSCG2020) ^(33)^. There are more specific guidelines, such as the “PADIS Guidelines” ^(34)^ for sedation, analgesia, and management of delirium, “Early Rehabilitation in Intensive Care: Evidence-Based Expert Consensus” ^(35)^, “Acute Kidney Injury (AKI) Clinical Guidelines 2016” ^(36)^, and the “Guidelines for Nutritional Therapy for Critically Ill Patients in Japan” ^(37)^.

COVID-19 drug therapies may include RNA polymerase inhibitors, immunomodulation therapy like steroid therapy to reduce the severity of the disease due to over-immune reactions, and anticoagulation to inhibit hypercoagulation. For the most recent recommendations on drug therapies for patients with COVID-19, please refer to the rapid/living recommendations for COVID-19 drug therapy ^(38)^.

The care of critically ill patients with COVID-19 at the end-of-life is not expected to be significantly different from other end-of-life care for critically ill patients. With regard to end-of-life care for critically ill patients, please refer to the “Guidelines for End-of-Life Care in Emergency and Intensive Care” ^(39)^ and “Practical Guide for End-of-Life Nursing” ^(40)^.

The COVID-19 pandemic has caused a collapse of medical care in other countries. Japan should also be prepared for the worst-case scenario, in which medical resources such as medical equipment, drugs, and human resources are exhausted. The Japanese Society of Intensive Care Medicine, in collaboration with the Japanese ECMOnet for COVID-19 and Research Group in Ministry of Health, Labor and Welfare Scientific Research Grant for “Research for Risk Assessment of Emerging and Re-Emerging Infectious Disease and Implementation of Risk Management Function,” has been working to develop the report “withholding and withdrawing of the treatment for major surge in coronavirus disease 2019 (COVID-19) pandemic, from the viewpoint of healthcare resource rationing” ^(41)^. Even in the worst-case scenario, where it is necessary to withhold or withdraw the treatment due to the limited medical resources, appropriate discussions should be held to ensure a fair supply of medical resources according to the principles of clinical ethics.

## Complications

### Coagulofibrinolytic change

Among patients with COVID-19, there are frequent thrombotic complications, such as deep vein thrombosis, pulmonary thromboembolism, cerebral infarction, and myocardial infarction due to vascular endothelial dysfunction and hypercoagulability, in addition to low activity associated with hospitalization and inflammation associated with infection [“COVID-19-associated thrombosis questionnaire findings” ^(42), (43), (44)^]. Patients with obesity or conditions such as cardiovascular disease and the elderly are thought to be at high risk of thrombotic complications. Therefore, thrombosis must be considered when treating these COVID-19 patients, and a testing and treatment plan needs to be established, which corresponds to the severity and risk of thrombotic complications.

Examples of blood-clotting tests in COVID-19 patients are platelets, D-dimer (FDP), fibrinogen, PT, and APTT. It is important to evaluate the changes in these tests over time, and keeping in mind the possibility of fluctuations within a short time, it is important to repeat measurements of routine assessments in a short time and repeat special tests as needed.

Thrombotic complications need to be considered when D-dimer suddenly rises during a patient’s course. If D-dimer is three to four times higher than the normal upper limit, the International Society on Thrombosis and Haemostasis guidelines recommend prophylactic administration of low-molecular-weight heparin (not approved in Japan) during hospitalization, even if there are no other symptoms. The following are also suggested in Japan:

1. In hospitalized patients with moderate or more severe disease, sharp increases in D-dimer or rapid deterioration of respiratory status are sometimes seen. Daily monitoring of D-dimer is recommended as needed.

2. In hospitalized patients with moderate or more severe disease, anticoagulant therapy with heparin should be considered with reference to D-dimer level and respiratory status.

3. Since DIC complications sometimes occur in severe cases, strict monitoring of such cases with blood-clotting tests is necessary.

4. Consider whether or not preventive anticoagulant therapy should be done after hospital discharge depending on the risk.

For the treatment of cardiovascular diseases, including myocardial infarction, please refer to the recommendations of the Japanese Circulation Society and the Japanese Association of Cardiovascular Intervention and Therapeutics ^(45), (46)^.

### Skin manifestations

Chilblain-like lesions may be the first manifestation of COVID-19, particularly in youth. These are associated with severe inflammation, a hypercoagulable state, and vascular endothelial injuries, although the exact pathogenetic mechanisms remain unclear. Livedo reticularis-like lesions may occur in patients with more severe respiratory symptoms. Histopathological evaluation of some skin biopsies showed thrombus formation and deposits of C5b-9 and C4d in venules, indicating complement pathway activation. These observations suggest that COVID-19 induces symptoms of cutaneous vasculitis.

### Acute kidney injury

Reports from multiple countries and regions demonstrated that a certain percentage of patients develop AKI during their clinical course of COVID-19. Furthermore, AKI is significantly related to patient mortality. Early recognition and treatment of AKI may improve the prognosis of patients with COVID-19. We recommend paying enough attention to AKI during the treatment of patients with COVID-19 ^(47)^.

### Heart failure

Direct damage to myocardial cells, systemic inflammation, and myocardial damage mediated by an excessive immune response in patients infected with SARS-CoV-2 have been suggested. Moreover, myocarditis and heart failure are often demonstrated by echocardiography and associated with serum biomarkers, such as BNP, NT-proBNP, and troponin I ^(48)^.

### Elevated blood glucose

Multiple reports have indicated associations between severity/prognosis of COVID-19 and hyperglycemia on admission or in hospital regardless of the presence or absence of diabetes mellitus. Treatment for COVID-19 including dexamethasone can cause elevation of blood glucose. In all COVID-19 cases, blood glucose levels should be monitored and managed appropriately ^(49)^.

### Sequelae

Recent studies from all over the world, including those performed in Japan, have reported long-term sequelae of COVID-19, attracting much attention. Common symptoms include fatigue, dyspnea, arthralgia, and chest pain and impaired taste and/or smell, hair loss, and sleep disturbance. These sequelae are attributed to complex pathogenic mechanisms associated with pulmonary or cardiac injury due to the cytokine storm, posttraumatic stress disorder such as severe anxiety and stress caused by isolation in the ICU, muscle weakness due to mechanical ventilation, and direct virus-induced injury ^(50)^. To date, no evidence-based therapeutic protocol has been established for the sequelae, and standard symptomatic therapies are usually used. Since patients show highly diverse symptoms, close cooperation among multiple departments is warranted to manage their physical and mental well-being continuously.

## Care for Special Situations: Care in Transplantation Process

When carrying out a transplantation, the risks and benefits should be considered and decided depending on the situation of COVID-19 in the area and in the hospital. Transplanted recipients are immunosuppressed and must be protected from donor-derived COVID-19, and the large number of medical staff involved in organ recovery and transportation of the graft must be prevented from exposure to COVID-19. The Japanese Society for Transplantation (JST) published and revised guidelines on special care of transplantation. JST distributes information on JST website ^(51)^ and updates it frequently.

In living donor transplantation, donors and recipients are directed to stay home or in the hospital for 14 days prior to transplantation, and immediate PCR and chest CT examinations are essential.

### (1) Transplant recipients

Transplant recipients have to visit the outpatient department for examination before prescribing immunosuppressive drugs. Thus, telemedicine is not possible, but JST recommends changing to a nearby transplant hospital or reducing the frequency of outpatient visits. Make sure that transplant patients and their families take action to prevent infection, do not discontinue immunosuppressive drugs at their own discretion for fear of infection, and contact by telephone instead of visiting the hospital directly when COVID-19 is suspected. It is necessary to consider in advance whether the transplant patient will be treated at his/her own hospital or requested to another hospital when he/she has COVID-19.

### (2) Prevention of COVID-19 from deceased donors after brain death/cardiac death

Although it is impossible to hear symptoms and exposure history from donors who do not speak for themselves, PCR tests are essential to fulfill their intention of providing organs and to ensure the safety of medical staff and transplant recipients, and they are accepted as governmental tests. The risk assessment of organ recovery from deceased donors is shown in the “Guidelines” ^(51)^.

### (3) “Mutual Aid System” for organ recovery from deceased donors after brain death/cardiac death

To minimize the movement of medical staff for organ recovery, organ recovery surgery is performed with the cooperation of a transplant hospital near the donor hospital, or if the donor hospital is a transplant hospital, the transplant doctors in that hospital recover the organs. Organ transport is carried out by a third party, including the Japan Organ Transplant Network or a cooperative company. This system is called the “mutual aid system,” and the examples are announced on JST website ^(51)^ for wider notification.

### (4) COVID-19 case registration of transplant patients

All COVID-19 transplant recipients have been registered and the outline is announced on JST website. The Task Force Committee Against COVID-19 of JST can provide advice on treatment for the registered case if requested.

## Care for Special Situations: Children

Pediatric patients account for a disproportionally small number of reported cases of COVID-19. Children show a lower incidence of symptomatic disease and rarely develop a severe course. Clinical presentation of COVID-19 in children is not characteristic and is indistinguishable from that of other respiratory infections. Lost or changed senses of smell and/or taste are among the most common symptoms of COVID-19 in adult patients; however, pediatric patients may not be able to complain if they experience this. Furthermore, children are often co-infected with more than one pathogen besides SARS-CoV-2.

A number of patients present with fever or respiratory symptoms in daily pediatric practice. Since it is difficult to distinguish them from those with COVID-19, we should collect epidemiological information as a clue to the diagnosis of COVID-19. It is critical to understand the epidemic and spread of COVID-19 in the neighborhood and know if he/she had contact with anyone who had fever or respiratory symptoms in his/her family, nursery, kindergarten, school, or neighborhood, since in the majority of children with COVID-19, it is transmitted from adults, especially their family members.

When collecting respiratory specimens from children, we should be aware of the following points: First, it is difficult to collect enough saliva from children. Second, it is also difficult for children to collect nasal swabs by themselves. Finally, when we try to collect specimens from them, they may weep and cry out, spraying doctors and other medical staff with droplets and aerosols. Therefore, we need to devise a method to collect nasopharyngeal swabs accurately and safely, avoiding exposure to their droplets and aerosols.

Case reports have appeared from abroad describing children with unusual systematic inflammatory conditions, termed multisystem inflammatory syndrome in children (MIS-C) or pediatric inflammatory multisystem syndrome (PIMS) temporally associated with SARS-CoV-2. MIS-C/PIMS occurs 2 to 4 weeks after infection with SARS-CoV-2, which can be asymptomatic or very mild and nonspecific, and most patients with MIS-C/PIMS have negative PCR at the time of symptom onset. Therefore, it may be difficult to make diagnosis of MIS-C/PIMS by involving COVID-19. No case of MIS-C/PIMS has been reported in Japan yet; however, suspected patients should be immediately referred to secondary/tertiary medical institutions considering its severity.

Most pediatric cases of COVID-19 are mild, requiring only observation or symptomatic treatment.

An increasing number of children have been complaining of mental problems due to school/nursery closure, abrupt changes in the social environment, and fear of illness. It is important to ensure the security and safety of children and support their guardians. Another concern is missed opportunities for vaccination and regular health checkups due to the COVID-19 pandemic. It is necessary to recommend guardians to bring their children for these essential activities in a timely manner while paying attention to infection ^(52), (53)^.

## Care for Special Situations: Pregnant Subjects

The frequency of COVID-19 in pregnant women is the same as that in non-pregnant women of the same age. Although the CDC lists pregnancy as one of the risk factors of poor prognosis ^(54)^, the rates of death and critically severe illness are not significantly increased in Japan.

The possible reasons for severe illness in mid- and late-gestational pregnant women are thought to be pulmonary compression due to diaphragmatic elevation and an increase in circulation volume, instead of a common misunderstanding of systemic immune suppression.

Fortunately, many reports have suggested that early pregnancy infections do not result in miscarriage or serious congenital anomalies, as observed in Zika fever and rubella. However, infection in the 3rd trimester often increases the rate of preterm births, mostly due to cesarean section indicated for maternal illness.

Intrauterine infections are very rare throughout the course of pregnancy, but some cases have been reported wherein IgM antibodies are present in the umbilical cord blood, suggesting intrauterine infection. Although there are no specific histopathological changes in the placenta, the presence of SARS-CoV-2-specific genome or viral proteins has been reported in placenta obtained from SARS-CoV-2-negative neonates. This fact suggests the possible presence of a placental barrier ^(55)^.

Management of COVID-19-infected patients in pregnant women is similar to that in normal adults, but favipiravir (Avigan) is absolutely contraindicated due to its strong teratogenicity. Due to its toxicity even after completion of the dose, abstinence is required for 2 weeks after completion of the dose, not only in patients receiving reproductive healthcare but also in men and women of reproductive age.

Aggressive anticoagulation should be used in pregnant women with COVID-19 infection due to their tendency to clot. Further, special attention should be given to patients with antiphospholipid antibody syndrome and gestational hypertension.

PCR screening of pregnant and nursing mothers should be performed considering the local infection status.

In principle, a Cesarean section is indicated to reduce the burden on the COVID-19 by shortening the delivery time and to prevent exposure of the medical staff, with the exception of cases where vaginal delivery is judged to be faster.

To reduce the risk of hospital-acquired infections, non-infected mothers are not allowed to chaperone, except for mentally ill people who cannot endure labor alone or if the patient requires a Japanese language interpreter. In these situations, her husband or other family members will be asked to undergo PCR screening before attendance.

Return home delivery will be determined by the attending physician depending on the prevalence of the disease in the area.

Considering the low risk of breast milk-borne infection, indirect breastfeeding from COVID-19 mothers through milking is possible if her condition is not serious and adequate attention is paid to the contamination. However, even in such cases, sufficient informed consent must be obtained from both parents ^(56)^.

## Gastrointestinal Endoscopy

Gastrointestinal (GI) endoscopy is a procedure with a high risk of generating droplets and aerosols. However, despite the current coronavirus disease (COVID-19) situation, it is considered possible to continue GI endoscopy, provided that protective measures are thoroughly implemented. Such measures may include the use of PPE, following assessment of the patient’s infection risk^(*NB)^ based on a prior medical interview and measurement of body temperature. Moreover, it should be carefully considered whether GI endoscopy is indicated prior to the procedure. In areas where the COVID-19 has spread, appropriate responses should be determined according to the prevailing national policies, local governments, and medical associations’ intentions.

The Japan Gastroenterological Endoscopy Society has issued recommendations ^(57)^ and clinical questions and answers ^(58)^ on COVID-19 and GI endoscopy. It is recommended to refer to these references when performing GI endoscopy.

(*NB) Patient risk assessment: It is recommended that, barring cases of medical emergencies, endoscopic treatment should be postponed in high-risk patients during the current COVID-19 situation. The following patients must be treated as high risk:

1) Patients presenting with any of the following: persistent cold symptoms, fever, breathing difficulties (dyspnea), and/or severe fatigue (malaise)

2) Patients with a recent history of close contact with cases of confirmed or suspected COVID-19 within the last 2 weeks

3) Individuals with abnormal sensations of taste and smell with no apparent cause

4) Individuals with gastrointestinal symptoms, including diarrhea persisting for 4-5 days with no apparent cause

## Mental Health (Especially for Patients and Healthcare Professionals)

The COVID-19 pandemic is classified as an extraordinary disaster in the category CBRNE (Chemical, Biological, Radiological, Nuclear, and high-yield Explosives). Compared to natural disasters, these extraordinary disasters have many uncertain factors and threats that are imperceptible; therefore, they tend to cause greater anxiety and fear and have a greater impact on daily lives such as commuting to work and school, resulting in high stress. Furthermore, self-isolation lifestyles, which require reduction in social and physical contact, can impede vital interpersonal interactions to maintain mental health, creating a strong sense of isolation and loneliness, and deprive them of various stress-relieving opportunities. In particular, mental health measures for infected persons, their families, and healthcare professionals are an urgent issue.

Infected persons are subject to a variety of psychological distresses, such as not only concerns about prognosis and aftereffects of the infection but also stress due to isolation, financial problems, anxiety about the possibility of having infected others, and discrimination and prejudice from those around them. It has also been suggested that COVID-19 can cause cognitive decline. When providing assistance to an infected person, it is prudent to fully understand that it is not at all unusual for an infected person to experience various psychological distress and then to consider measures and reach out to a specialist.

In the case of healthcare professionals, in addition to the anxiety that they may become infected while performing their duties, they are also placed in a situation where they are more likely to feel the moral injury of having to deal with medical care and treatment of COVID-19 with limited resources and information. There is also a possibility that healthcare professionals will be subject to discrimination from their surroundings. It is important to take more care of yourself than usual, such as getting enough rest and sleep during work and shifts, eating a healthy diet, exercising, and keeping in touch with family and friends. Moreover, it is important to actively seek support from superiors, colleagues, and trusted people as needed, without feeling embarrassed about seeking support.

In addition, managers and team leaders of organizations need to have more mindful communication with staff, update accurate information, and fix flexible schedules more than usual. In addition to taking care of oneself, encouraging staff to take care of themselves and providing mutual support may also contribute to the mental health of each staff and the organization.

The Japanese Society of Psychiatry and Neurology provides a collection of links that summarize useful information in Japan and overseas^(59)^.

The Guidelines for Mental Health Measures under the Novel Coronavirus (COVID-19) Pandemic^(60)^ summarizes mental health support for individual workers, infected persons, quarantined individuals, bereaved families, healthcare professionals, children, parents, elderly persons, pregnant women, and students.

## Return to Work Criteria (Work and School, Healthcare Settings)

It has been reported that patients with COVID-19 are most contagious 2 days before symptom onset^(61)^ to the onset day and the infectivity is estimated to decline within 7 days after symptom onset^(61)^. The governmental notification^(62)^ issued on June 25, 2020, no longer requires any PCR test before the discharge from hospitals and the ending isolation for the lifting of work restrictions based on Article 18 of the Infectious Diseases Control Law. It says that work restriction can be lifted once “10 days have passed since symptoms onset AND 72 h have passed since resolution of fever.” Routine and standard infection prevention and control, such as wearing a face mask, hand hygiene, physical distancing, and adequate ventilation, are recommended in workplaces and school rooms.

The guidance for the person with COVID-19 at returning to work or school is summarized in Table 2.

**Table 2.** Guidance for Returning to Work or School.

Work and school（non-healthcare settings）	Healthcare settings
Persons with COVID-19 may discontinue isolation under the following conditions:	
At least 10 days have passed since symptom onset (or after the date of first positive COVID-19 test)	
At least 72 h have passed since resolution of fever^(*1)^ and other symptoms have improved^(*2)^	
Person should seek advice from infection control experts, occupational physicians, school physicians, etc., prior to returning to work or school	
（*1）Without the use of antipyretics	
（*2）Symptoms such as cough/fatigue and respiratory distress (loss of taste or smell might be long term in COVID-19 patients)	
In case of severe or hospitalized person with COVID-19, they should consider consultation with infection control expert to ensure safe returning to work or school.	In addition to remarks of non-healthcare settings, consider the following measures as well.
After returning to work or school, routine infection prevention and control such as daily self-monitoring for symptoms, wearing a face mask, and physical distancing should be implemented as usual.	Healthcare professionals who take care of vulnerable patients may return to work 20 days after the onset of symptoms ^(64)^ or two consecutive negative PCR tests ^(65)^.

Special considerations should be taken to ensure protection from discrimination or prejudice against not only persons with COVID-19 but also their families, colleagues, or healthcare professionals ^(66)^.

It is also necessary to make people in the workplace and school understand not to practice harassment, bullying, encouragement to retire, and stigma or slander on SNS derived from rumors.

Especially at schools, it is necessary to consider that parents should not be involved in any information from children and students, who have limited information and knowledge.

If a person has fever or flu-like symptoms, they should be strongly advised to contact healthcare providers to seek medical advice on needs for COVID-19 test as much as possible. If you are a healthcare professional, you should make sure to provide a COVID-19 test. If the person cannot take a COVID-19 test (including those who have not visited a medical provider), they are deemed to be a person with COVID-19. Therefore, we have summarized the guidance for returning to work or school in Table 3.

**Table 3.** Guidance for Returning to Work or School (for Those Who Have Fever or Flu-like Symptoms but Have Not Been Diagnosed with COVID-19).

Non-healthcare settings
Persons may discontinue isolation under the following conditions:
At least 8 days have passed since symptom onset
At least 72 h have passed since resolution of fever^(*1)^ and other symptoms have improved^(*2)^
If it is difficult to take a leave of absence during the above period due to urgent business reasons, COVID-19 test should be recommended as much as possible. If it is not possible to have a COVID-19 test, each business should implement the following conditions at their own risk.
At least 72 h have passed since the fever and symptoms have improved^(*1)^
（*1）Without the use of antipyretics
（*2）Symptoms such as cough/fatigue and respiratory distress
After returning to work or school, routine infection prevention and control such as daily self-monitoring for symptoms, wearing a face mask, and physical distancing should be implemented as usual.
This expectation does not apply in the cases limited to “working from home.” However, they should pay much attention to risk of infection in households.

The Japanese Society for Occupational Health has published in collaboration with the Japanese Society of Travel and Health “The Guidance on Preparing Workplaces against spread of COVID-19” ^(67)^.

Other information is also available, such as “ventilation simulator for COVID-19” in our website ^(67), (68)^.

## Article Information

### Conflicts of Interest

None

### Author Contributions

Takashi Kadowaki served as a supervisor. All the other authors wrote chapters and checked the contents mutually.

### 

This is the English version of the JMSF COVID-19 expert opinion published on the JMSF homepage on November 20, 2020 ^(69)^. The original Japanese version is no longer available. This submission is approved by JMSF and Editor-in-Chief of JMA Journal.
